# Hand‐Specific Engagement of Cerebello–Thalamo–Cortical and Higher‐Order Sensorimotor Networks in Essential Tremor: Converging Evidence From GLM and MVPA‐Based fMRI Analysis

**DOI:** 10.1111/ene.70662

**Published:** 2026-06-14

**Authors:** Alma S. Torres‐Torres, Jelle R. Dalenberg, A. M. Madelein van der Stouwe, Bauke M. de Jong, Remco J. Renken, Giorgia Sciacca, Marina A. J. Tijssen

**Affiliations:** ^1^ Department of Neurology University Medical Center Groningen Groningen the Netherlands; ^2^ Cognitive Neuroscience Center, Department of Biomedical Sciences of Cells and Systems University Medical Center Groningen Groningen the Netherlands; ^3^ Department of Medical, Surgical Sciences and Advanced Technologies G. F. Ingrassia University of Catania Catania Italy

**Keywords:** BOLD signal decoding, cerebello‐thalamo‐cortical network, motor lateralization, searchlight analysis, sensorimotor integration, thalamic dysfunction

## Abstract

**Background:**

Essential tremor (ET) is a prevalent movement disorder affecting the upper limbs, yet the neural basis of hand‐specific motor dysfunction remains incompletely understood. Most fMRI studies have not explicitly addressed lateralization of tremor‐related brain activity. This study used both multivariate pattern analysis (MVPA) and univariate general linear modeling (GLM) to characterize brain network engagement during right‐ and left‐hand movements in ET.

**Methods:**

Eighteen right‐handed ET patients and 18 age‐matched healthy volunteers performed alternating right‐ and left‐hand finger‐tapping tasks during fMRI. Whole‐brain GLM assessed subject‐level BOLD activation, and searchlight MVPA quantified subject‐level local decoding performance. Group‐level analyses compared activation and decoding patterns between groups, and associations with hand‐specific tremor severity were examined within the ET group.

**Results:**

During right‐hand finger‐tapping, ET patients exhibited bilateral motor network engagement. GLM revealed reduced BOLD activity in the thalami and right supramarginal gyrus, while MVPA showed decreased decoding in the right supramarginal gyrus. In left‐hand finger‐tapping, a more lateralized pattern emerged, with diminished activation and decoding performance in the right thalamus and right supramarginal gyrus. Left cerebellar involvement differed between tasks and showed putative compensatory engagement, and right insular activity was associated with tremor severity during task performance. Overall, cortical alterations were predominantly right lateralized across both hands.

**Conclusions:**

ET is associated with hand‐specific differences in task‐related network engagement, involving cerebello–thalamo–cortical and higher‐order sensorimotor regions. Right‐hand movements engaged more bilateral motor circuits, whereas left‐hand movements showed relatively stronger right‐hemispheric involvement, suggesting lateralized task‐related network alterations in ET.

## Graphical abstract

Essential tremor (ET) was investigated using task‐based fMRI during right‐ and left‐hand finger‐tapping in combination with univariate GLM and searchlight MVPA. ET patients exhibited hand‐specific differences in cerebello–thalamo–cortical and higher‐order sensorimotor engagement, with reduced thalamic and right supramarginal involvement compared to healthy volunteers. Right‐hand movements engaged more bilateral motor circuits, whereas left‐hand movements showed relatively stronger right‐hemispheric involvement.

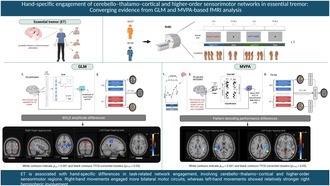

**Abbreviations**
BOLD: blood‐oxygen‐level‐dependent
DBS: deep brain stimulation
ET: essential tremor
fMRI: functional magnetic resonance imaging
FTM‐TRS: Fahn‐Tolosa‐Marin Tremor Rating Scale
GLM: general linear model
HADS: Hospital Anxiety and Depression Scale
HV: healthy volunteers
LTS: left tremor severity
M1: primary motor cortex
MoCA: Montreal Cognitive Assessment
MVPA: multi‐voxel pattern analysis
RTS: right tremor severity
SMA: supplementary motor area
UMCG: University Medical Center Groningen

## 1 Introduction

Essential tremor (ET) is one of the most prevalent movement disorders, affecting approximately 1.3% of the global population [1]. It is characterized by involuntary oscillatory action tremor in the upper limbs [2] and can significantly impair quality of life, particularly when tremor severity interferes with daily activities such as writing, eating, and drinking [3]. Diagnosis primarily relies on clinical examination focused on the exclusion of other tremor syndromes identified by additional signs and symptoms [4], yet misdiagnosis rates range from 30% to 50% [5] due to symptom heterogeneity and the absence of definitive biomarkers [6]. These limitations hinder accurate diagnosis and motivate studies aimed at improving our understanding of the underlying pathophysiology.

Functional magnetic resonance imaging (fMRI) is a valuable tool for probing the neural mechanisms of ET by measuring task‐related changes in blood‐oxygen‐level‐dependent (BOLD) signals [7]. In the context of ET, task‐based fMRI captures neural activity related to voluntary motor execution performed in the presence of superimposed tremor, rather than isolating tremor‐related activity per se. Previous task‐based fMRI studies comparing ET patients with healthy volunteers (Table 1) have consistently implicated abnormalities within the cerebello‐thalamo‐cortical network, particularly involving the primary motor cortex (M1), cerebellum, and supplementary motor area (SMA). However, findings have been inconsistent: while some studies report cerebellar overactivation [8], others have observed reduced cerebellar activation [9, 10, 11, 12, 13]. Similarly, activity in the SMA and M1 has been reported as both increased [11, 12] and decreased [10]. These discrepancies likely stem from variability in patient phenotypes, task paradigms, and analytic approaches.

**TABLE 1:** Task‐based fMRI studies in ET.

Year	Groups	Age	Upper limb paradigm	Task(s)	Analysis	Highlights	References
2022	ET 14	59.6 ± 18.2	Dominant (right)	Wrist motor task	GLM	Sensorimotor network function in ET did not differ from HV. Results indicate involvement of the ION in ET tremor modulation. Activity in the bilateral CBM, showed a positive correlation with tremor severity	[14]
	HV 18	55.4 ± 13.1					
2020	ET 16	70.3 ± 8.1	Dominant (right)	Maintaining a posture (DBS on/off)	GLM	The reduction in tremor induced by DBS is associated with decreased motor activity in the M1, CBM, and SMA during stimulation compared to no stimulation	[15]
2020	ET 10	69.4 ± 8.9	Dominant (right)	Maintaining a posture	GLM	Decreased activity in CBM, sensory‐motor cortex, and BG in ET while maintaining a posture compared with HV	[11]
	HV 10	67.7 ± 7.8					
2018	ET 19	65.7 ± 11.6	Dominant (right)	Force task (visual feedback)	GLM	Decreased activity in CBM, M1, SPG and IPG and LG compared with HV, while BOLD entropy in V3 and V5 showed a positive correlation with tremor severity	[12]
	HV 18	63.7 ± 7.6					
2015	ET 21	51.6 ± 17.8	Dominant (right)	Maintaining a posture	GLM	Tremor‐associated bilateral increased cerebellar activity compared to mimicked tremor in HV	[10]
	HV 21	50.6 ± 16.4					
2015	ET 31	55.4 ± 15.8	Dominant (right)	Rhythmic finger tapping	GLM	Decreased activity in CBM, parietal and frontal cortex, DN and ION in ET compared to HV. Positive correlation between activity in bilateral DN and tremor severity	[16]
	HV 29	52.6 ± 16.1					
2015	ET 14	61.7 ± 11.0	ET: Most tremulous HV: Dominant	Force task	GLM	Increased activity in M1 and SMA compared with HV, which correlated positively with 3–8 Hz force oscillations. Brain activity in CBM I–V was reduced in ET compared with HV and correlated negatively with 0–3 Hz force oscillations	[13]
	HV 14	60.2 ± 9.2					
2015	ET 32	69.7 ± 9.7	Dominant (right)	Writing of “8”	GLM	Decreased activity in CBM, thalamus, and parietal cortex, while PMC, SMA, precuneus, and SPG showed increased activity during motor tasks in ET compared to HV	[17]
	HV 12	67.4 ± 4.8					

Abbreviations: BG, basal ganglia; BOLD, blood‐oxygenation level dependent; CBM, cerebellum; DBS, deep brain stimulation; DN, dentate nucleus; ET, Essential tremor; GLM, general linear model; HV, healthy volunteers; ION, inferior olive nucleus; IPG, inferior parietal gyrus; LG, lingual gyrus; M1, primary motor cortex; PMC, posterior medial cortex; SC, superior colliculus; SPG, superior parietal gyrus; SMA, supplementary motor area; V3, visual area three; V5, visual area five.

The thalamus, a key relay structure in motor control, plays a central role in cerebello–thalamo–cortical communication and tremor generation [16, 17], yet remains relatively underreported in task‐based fMRI studies of ET. Only one task‐based study to date has reported reduced thalamic activity in ET patients [12], highlighting the heterogeneity in thalamic findings across the literature.

Several task‐based fMRI studies have linked tremor severity to alterations in brain activity in ET. Increased cerebellar activity, including bilateral hemispheres and the dentate nucleus, has been shown to correlate positively with tremor severity during motor tasks [13, 18]. However, although tremor severity is frequently assessed clinically, hand‐specific tremor asymmetry is rarely incorporated explicitly into task‐based fMRI analyses, despite its relevance to motor symptom expression and underlying network dynamics [19].

Most previous fMRI studies in ET have focused exclusively on right‐handed individuals performing dominant‐hand tasks, with only one accounting for the side of greater tremor severity by flipping imaging data [11]. However, such approaches risk obscuring relevant lateralized neural differences. Historically, most fMRI studies have used univariate analyses like general linear modeling (GLM) to detect task‐related activation. In contrast, multivariate approaches such as multivariate pattern analysis (MVPA) decode distributed neural patterns, offering complementary insights into brain function. By capturing fine‐scale activity and voxel interdependencies, MVPA provides increased sensitivity for detecting network‐level alterations associated with ET [20].

In this study, we analyzed left‐ and right‐hand motor tasks separately in right‐handed ET patients and matched right‐handed healthy volunteers to investigate task‐related lateralization of motor network engagement and ipsilateral contributions. We explicitly distinguish between task‐related lateralization (left‐ versus right‐hand motor execution) and clinical asymmetry of tremor severity, which represent partially independent phenomena and are both relevant for understanding ET pathophysiology [14]. Our primary objective was to characterize differences in brain network engagement between the right and left upper limbs in right‐handed participants and to examine how hand‐specific tremor severity relates to neural activity, thereby capturing aspects of clinical heterogeneity. To this end, we used whole‐brain univariate GLM and searchlight‐based MVPA to estimate subject‐level BOLD activation and decoding performance. These subject‐level metrics were subsequently entered into second‐level group analyses to compare ET patients and healthy volunteers, while tremor severity was examined within the ET group using hand‐specific measures. By characterizing hand‐specific and lateralized patterns of network engagement, this study provides network‐level insights into the lateralized pathophysiology of ET and may inform future hypothesis‐driven investigations of targeted therapeutic strategies.

## 2 Materials and Methods

### 2.1 Participants

For the current study, we included a total of 18 right‐handed ET patients and 18 age‐matched right‐handed healthy volunteers from the Next Move in Movement Disorders (NEMO) study [15]. The NEMO protocol is a standardized framework for phenotyping hyperkinetic movement disorders, combining structured clinical evaluation and validated rating scales, with diagnosis confirmed by expert consensus. This framework ensures consistent patient enrollment and standardized assessment of tremor severity across participants. All participants were right‐handed, ensuring consistent interpretation of right‐ and left‐hand motor tasks across individuals. Patients were identified through the University Medical Center Groningen's (UMCG) hyperkinetic movement disorders database and were enrolled at the UMCG outpatient clinic. Inclusion was based on the diagnosis of essential tremor made by the treating movement disorder specialist, in accordance with the current consensus criteria for ET [21]. During the study visit, a structured interview included questions about age at onset, family history, handedness, and self‐reported alcohol responsivity. Additionally, the Hospital Anxiety and Depression Scale (HADS) questionnaire was used to assess anxiety and depression in all participants; the Montreal Cognitive Assessment (MoCA) was used to measure cognitive impairment in all participants.

Tremor severity was assessed within the NEMO framework using the Fahn–Tolosa–Marin Essential Tremor Rating Scale (FTM‐TRS) [22] by an independent movement disorders expert. Hand‐specific tremor severity scores were derived separately for the right and left hand for use in subsequent analyses. The NEMO study protocol received approval from the Medical Ethical Committee (METc 2018–444) of the UMCG and written informed consent was obtained from all subjects according to the Declaration of Helsinki.

### 2.2 Finger‐Tapping Tasks

The experimental design incorporated a finger‐tapping task of each hand. Participants performed three distinct tasks within the MRI scanner: (1) right‐hand finger‐tapping task, in which participants tapped all four right fingertips simultaneously against the thumb in a repetitive motion for 10 s, while the left hand remained at rest in a supine position; (2) left‐hand finger‐tapping, in which participants tapped all four left fingertips simultaneously against the thumb in a repetitive motion for 10 s, while the right hand remained at rest in a supine position; and (3) rest, during which participants were instructed to keep both hands at rest in a supine position for randomly jittered intervals ranging from 15 to 20 s.

Right‐ and left‐hand finger‐tapping conditions were modeled separately to enable within‐subject assessment of task‐related lateralization. Tasks 1 and 2 alternated as shown in the Figure S1, and this block of tasks was repeated five times.

Instructions for each task were displayed on a screen located behind the scanner, which participants could see via a mirror attached to the head coil. The entire session was recorded over an approximate duration of 5 min. To ensure correct task performance, we showed an instruction video before the scan and let the participant practice in the scanner before measurements began. Task performance was continuously monitored via video recording during scanning, and datasets showing incorrect task execution or excessive voluntary movement were excluded from further analysis.

No objective behavioral measures (e.g., tapping frequency, error rates) or physiological recordings (e.g., accelerometry or EMG) were acquired during scanning; therefore, task‐related neural activity reflects voluntary motor execution performed in the presence of superimposed tremor rather than isolated tremor‐related activity.

### 2.3 fMRI Data Acquisition and Artifact Removal

A detailed description of the NEMO imaging protocols is available in [23]. In short, MRI data were collected on a 3T Siemens Prisma scanner at the UMCG using a Siemens 64‐channel head coil. Task‐based fMRI scans were acquired using a multiband, multi‐echo T2*‐weighted echo‐planar imaging (EPI) sequence with the following scanning parameters: TR = 1.101 s; TE = 12, 36.1, 60.2 ms; voxel size = 3.5 mm isotropic. The EPI pulse sequence used was provided by the Center for Magnetic Resonance Research (CMRR) at the University of Minnesota [24]. To reduce motion‐related artifacts, data were denoised using a previously validated preprocessing pipeline for NEMO [23], incorporating fMRIPrep (v22.0.2) [25], tedana (v0.0.12) [26], and ANTs (v2.3.5) [27]. Specifically, the use of multi‐echo fMRI combined with ME‐ICA denoising enables data‐driven separation of BOLD and non‐BOLD components based on echo‐time dependence, providing robust removal of motion‐related and task‐correlated artifacts beyond standard motion parameter regression [28]. The resulting denoised BOLD time series was used as input for a GLM analysis and searchlight MVPA.

### 2.4 General Linear Model (GLM) Analysis

Our data analysis includes employing the GLM approach to compare our findings with those reported in the existing literature and as a complement to MVPA, with MVPA being more sensitive to distributed coding and GLM to global task engagement [20].

To obtain the brain map of the BOLD response distribution for each participant under each condition, we conducted a subject‐level analysis using Nilearn (v0.10.4). The regressors included each finger‐tapping task, the realignment parameters, and their first derivatives estimated by fMRIPrep as covariates. In this model, neural activations related to right‐ and left‐hand finger‐tapping tasks were modeled using the rest windows as the baseline. The onset and duration of each finger‐tapping task were convolved with a Glover hemodynamic response function (HRF) to predict the expected BOLD response.

For within‐ and between‐group comparisons, group‐level contrast images were generated with age included as a covariate. Additionally, we explored how tremor severity influenced brain activity in ET patients by including hand‐specific clinical tremor severity scores as between‐subject regressors at the group (second‐level) analysis, within the ET group, with age included as a covariate. Accordingly, associations with tremor severity reflect interindividual variability in clinical tremor severity among ET patients and its relationship to task‐related neural engagement.

In addition, an exploratory whole‐brain Group × Hand interaction analysis (ET vs. HV × Right vs. Left hand) was performed at the second level using permutation testing with TFCE correction.

### 2.5 Searchlight Multi‐Voxel Pattern Analysis (MVPA)

Searchlight MVPA improves the detection of localized brain activity patterns by analyzing small regions around each voxel, which helps in identifying task‐relevant response patterns [29].

We analyzed our task‐fMRI data using searchlight MVPA to generate subject‐level decoding accuracy maps and compare these on a group level. This analysis was set up using BrainIAK v0.11 to classify conditions of right‐hand finger tapping versus rest and left‐hand finger tapping versus rest for each participant. MVPA analyses were used to characterize distributed, hand‐specific task‐related activation patterns at the network level rather than to classify individuals or identify diagnostic biomarkers. MVPA decoding performance therefore reflects differences in distributed neural patterns associated with task execution under tremor conditions rather than dissociating voluntary movement from tremor‐related neural activity.

The implementation of lags in our study aimed to monitor classification changes across different time frames, accounting for the BOLD response's slower dynamics relative to the rapid timescale of neural activity (hemodynamic response).

**FIGURE 1:**
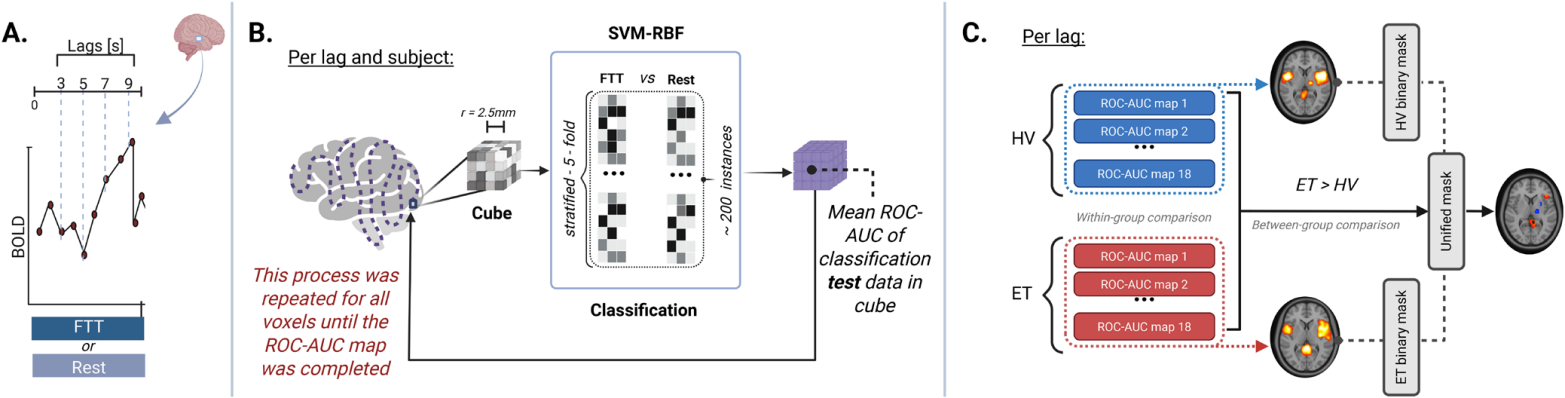
Schematic overview of the searchlight multi‐voxel pattern analysis (MVPA) procedure applied to distinguish Essential tremor (ET) patients, from healthy volunteers (HV) in right and left finger‐tapping tasks. (A) Employing four poststimulus time‐lags at intervals of 3, 5, 7, and 9 s. (B) The searchlight MVPA analysis was conducted using 5 × 5 × 5 (125) voxel cubes, applying radial basis function (RBF) SVM within a stratified 5‐fold cross‐validation framework, this process generated a cross‐validation ROC AUC map for each subject and time lag. (C) Within‐ and Between‐group fMRI analyses on MVPA‐based decoding performance maps.

We evaluated decoding performance for both right‐ and left‐hand finger‐tapping tasks at four poststimulus time lags (3, 5, 7, and 9 s), as illustrated in Figure 1A, to account for the delayed HRF relative to neural activity. These lags were used to capture early and late phases of the task‐evoked BOLD signal.

The searchlight MVPA analysis, shown in Figure 1B, was performed using 5 × 5 × 5 (125) voxel cubes to minimize loss of decoding information [30]. A radial basis function (RBF) SVM was applied within a stratified 5‐fold cross‐validation framework (scikit‐learn v1.1.0). Each participant's dataset comprised approximately 200 volumes, including ~100 rest volumes, ~50 right‐hand finger‐tapping volumes, and ~50 left‐hand finger‐tapping volumes. Multivariate analyses were performed using five‐fold cross‐validation, such that in each fold approximately 80% of the data (~160 volumes) were used for training and 20% (~40 volumes) were used for testing. Data were stratified within each fold to preserve balanced proportions of rest and finger‐tapping conditions across training and testing sets, and samples were assigned to folds such that no overlap occurred between training and testing data. Each test fold therefore contained approximately 40 volumes, proportionally representing all experimental conditions.

This process, shown in Figure 1B, utilized the mean area under the receiver operating characteristic curve (ROC AUC) as a measure of MVPA‐based decoding performance. Higher values indicate greater ability to distinguish task from rest conditions, reflecting more consistent and task‐specific local BOLD patterns. Lower values indicate more variability or overlap in activation across conditions.

To identify task‐relevant response patterns within and between healthy volunteers and ET patients, both within‐group and between‐group comparisons were performed (Figure 1C). Group‐level comparisons were performed with age included as a covariate. As in the GLM analysis, tremor severity was modeled at the between‐subject level and not as a time‐varying regressor. These analyses therefore capture tremor‐severity–related differences in task‐related neural patterns rather than tremor dynamics during task execution.

### 2.6 Statistical Comparisons

#### 2.6.1 Demographic Distribution

Differences between ET patients and healthy volunteers in gender were assessed using chi‐square (*χ* ^2^) tests; age, MoCA, and HADS scores with independent *t*‐tests. Paired *t*‐tests evaluated tremor severity asymmetry between right‐ and left‐hand tremor severity scores in ET patients.

#### 2.6.2 Within‐ and Between‐Group fMRI Contrasts

We used GLM to identify BOLD activation patterns in ET and healthy volunteers (HV), including group comparisons and associations with tremor severity. Within‐group effects were tested using two‐sided permutation tests (10,000 permutations) in Nilearn (v0.10.4).

MVPA was used to assess distributed neural activity differences using subject‐level decoding maps. One‐sided permutation tests were used to identify within‐group effects, followed by two‐sided permutation tests for between‐group comparisons. To ensure robust interpretation of between‐group effects, analyses were restricted to a data‐driven unified mask derived from significant within‐group decoding results (Figure 1C). Between‐group results are presented within this unified mask for interpretational clarity. Importantly, this unified mask was applied post hoc and was not used to constrain whole‐brain statistical testing. Accordingly, reported between‐group differences reflect variations in regions demonstrating reliable decoding performance within each group independently, rather than effects driven by chance‐level decoding.

Threshold‐Free Cluster Enhancement (TFCE) was applied to correct for multiple comparisons, which was selected for its superior sensitivity in detecting relevant clusters [31]. Statistical inference for between‐group contrasts in both GLM and MVPA analyses was based on TFCE‐corrected results at *p*_*TFCE*_ of < 0.05. For visualization and reporting purposes only, clusters exceeding an uncorrected voxel‐wise threshold of *p*_*unc*_ < 0.001 were additionally described.

## 3 Results

### 3.1 Participants

The study included 18 ET patients and 18 healthy volunteers. Initially, 19 patients participated, of whom one was excluded due to a technical issue while recording the tapping task. The demographic and clinical characteristics of the participants are summarized in Table 2. The mean age did not differ between ET patients (65.0 (±14.78)) and healthy volunteers (62.5 (±12.71)) (*t* = 0.55, *p* = 0.581). Similarly, there was no difference in male/female distribution between groups.

**TABLE 2:** Demographic and clinical characteristics of study participants.

	ET	HV	Statistical value	*p*
*N*	18	18		
Age (years)	65.0 ± 14.78	62.5 ± 12.71	*t* = 0.557	0.581
Male/female	13/5	10/8	*χ* ^2^ = 0.482	0.482
HADS	4.47 ± 4.77	5.01 ± 4.11	*t* = 0.354	0.725
Depression	2.44 ± 2.83	1.71 ± 2.29	*t* = 0.079	0.948
Anxiety	2.28 ± 2.21	2.84 ± 2.41	*t* = 0.944	0.419
MoCa	26.89 ± 1.49	27.49 ± 2.49	*t* = 0.741	0.465
Age at onset (years)	36.2 ± 24.81	—	—	—
FTM‐TRS A	6.7 ± 3.88	—	—	—
RTS	3.1 ± 2.29	—	—	—
LTS	3.7 ± 2.01	—	—	—
Alcohol responsivity (PR/NR)	5/13	—	—	—

Abbreviations: ET, Essential tremor; FTM‐TRS‐A, Fahn Tolosa Marin Essential Tremor Scale (mean *±* standard deviation); HADS, Hospital Anxiety and Depression Scale (mean ± standard deviation); HV, Healthy volunteers; LTS, left‐hand tremor severity; MoCA, Montreal Cognitive Assessment, Age in years (mean ± standard deviation); *N*, Number of participants; NR, no response; PR, positive response; RTS, right‐hand tremor severity.

Moreover, we found no significant differences between ET and healthy volunteers regarding HADS depression and anxiety scores. Cognitive function, as measured by the MoCA, did not differ between ET (26.89 (±1.49)) and healthy volunteers (27.49 (±2.49)) (*t* = 0.741, *p* = 0.465). These results indicate demographic similarities between the groups, allowing meaningful comparisons in subsequent fMRI analyses.

In the ET group, the age at tremor onset was 36.2 years (±24.81). The mean FTM‐TRS‐A score was 6.8 (±3.72). Hand‐specific differences in tremor severity between hands were observed in 14 ET patients, with left‐hand tremor severity (LTS) greater than right‐hand tremor severity (RTS) in 9 patients (Figure S2). Overall, the mean RTS score was 3.2 (±2.27) and the mean LTS score was 3.6 (±1.98), with no statistically significant difference between hands (*t* = 1.24, *p* = 0.23). Regarding alcohol responsivity, 5 patients in the ET group reported a positive alcohol response, while 13 reported no alcohol suppression.

### 3.2 GLM Results

**FIGURE 2:**
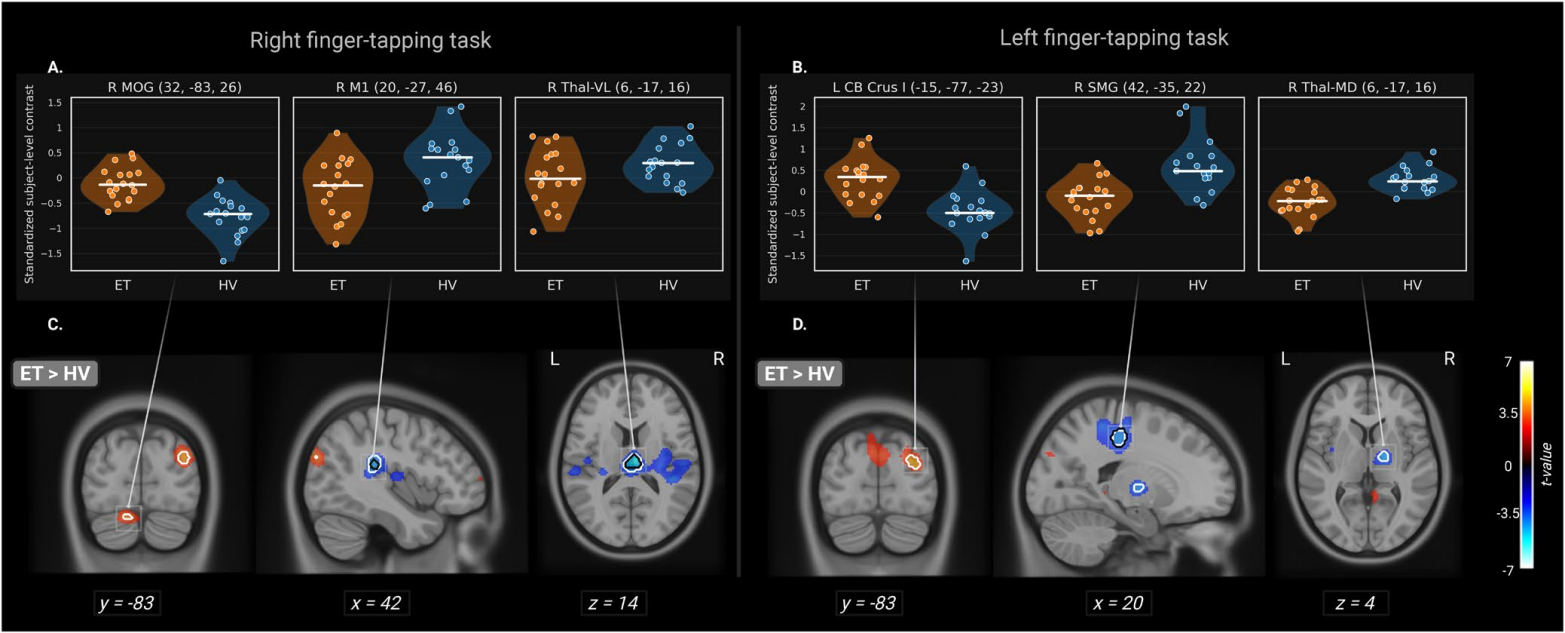
Task‐related BOLD responses during finger‐tapping in essential tremor (ET) and healthy volunteers (HV). (A, B) ROI‐level violin plots with overlaid scatter points showing standardized subject‐level contrast estimates for regions exhibiting significant between‐group differences during the right (A) and left (B) finger‐tapping tasks. Dots represent individual participants; white bars indicate group means. ROI names and peak MNI coordinates are shown above each plot. (C, D) Whole‐brain GLM maps for the between‐group contrast (ET > HV) during the right (C) and left (D) finger‐tapping tasks, thresholded at *p*_*unc*_ < 0.01. White contours indicate *p*_*unc*_ < 0.001 and black contours TFCE‐corrected clusters (*p*_*TFCE*_ < 0.05). Blue colors denote reduced BOLD responses in ET relative to HV; red colors denote increased responses. Slice locations are shown in MNI space.

**FIGURE 3:**
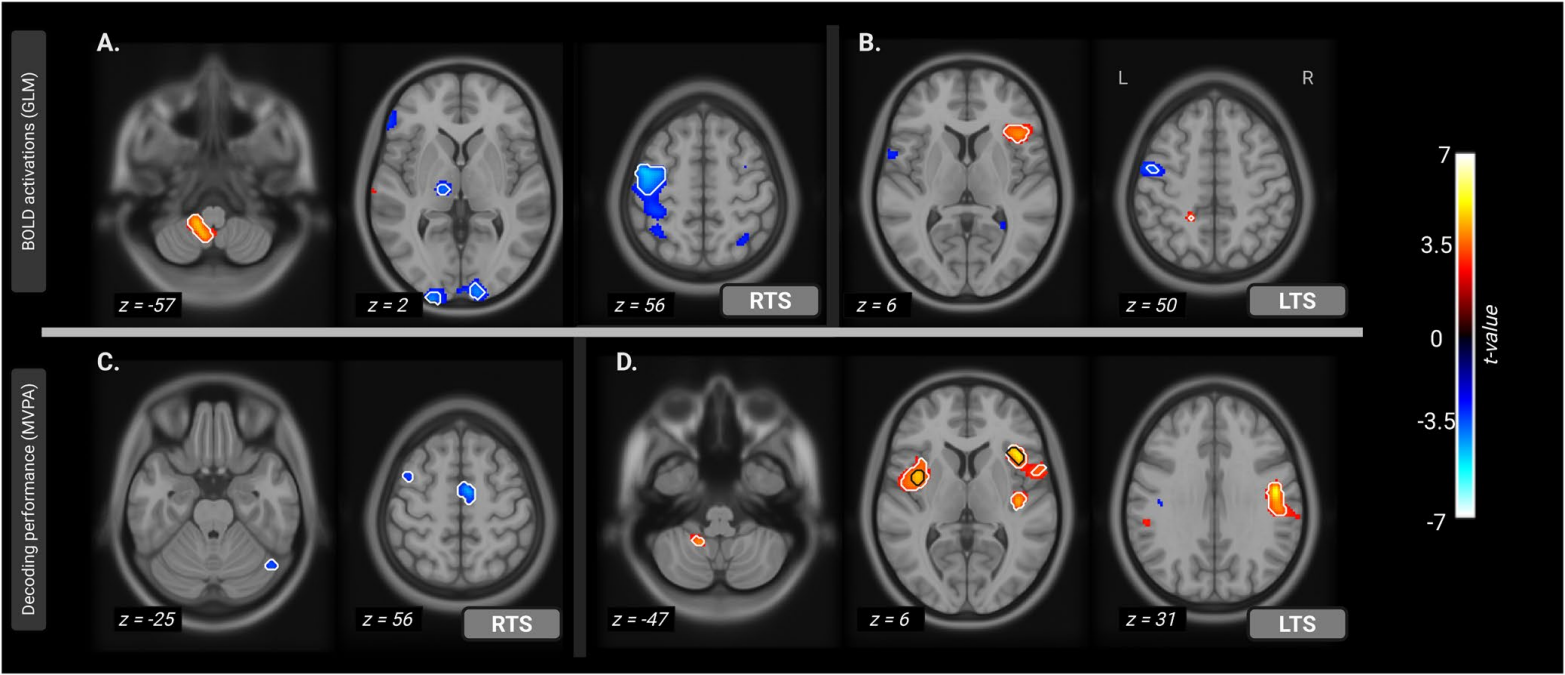
Brain regions associated with tremor severity using GLM analysis of BOLD activations (top row, A, B) and decoding performance using MVPA (bottom row, C, D). Warm colors (red‐yellow) indicate positive associations, while cool colors (blue) indicate negative associations. Results are thresholded at *p*_*unc*_ < 0.01, with additional contours indicating stricter statistical thresholds: At *p*_*unc*_ < 0.001 (white contour) and *p*_*TFCE*_ < 0.05 (black contour). (A, B) Show BOLD activations correlated with tremor severity for RTS (right tremor severity) and LTS (left tremor severity), respectively. (C, D) depict MVPA decoding performance for RTS and LTS.

GLM results show BOLD response patterns within and between groups. In healthy volunteers, the paradigm activated the motor network (Figure S3). Group differences for right and left finger‐tapping are shown in Table 3 and Figure 2, with tremor severity associations in Figure 3A,B.

**TABLE 3:** GLM and MVPA between‐group comparison of brain regions in right‐ and left‐finger‐tapping task.

	Finger tapping task	*N*	Contrasts		Brain region	X	Y	Z	*t*‐value	*p*_*unc*_	Cluster size [mm^3^]	*p*_*TFCE*_
GLM	Right	1	ET > HV	L	Cerebellum crus I	−15	−77	−23	4.97	2.2E−05	944	—
		2	ET > HV	R	Middle occipital gyrus	38	−83	28	4.06	7.1E−04	552	—
		3	ET < HV	R	Thalamus—mediodorsal	6	−17	16	5.72	1.0E−06	5072	0.0028
		3b	ET < HV	L	Thalamus—pulvinar	−2	−27	4	3.69	7.1E−04		0.0188
		4	ET < HV	R	Supramarginal gyrus	42	−35	22	4.49	7.5E−05	1160	0.0171
		5	ET < HV	L	pSTG	−33	−29	10	3.78	7.2E−04	64	—
		6	+RTS	L	Cerebellum IX	−14	−45	−57	5.01	1.0E−04	688	—
		7	+RTS	R	Postcentral gyrus (SMA)	17	−40	79	4.45	3.6E−04	320	—
		8	−RTS	L	Precentral gyrus (M1)	−46	−5	56	4.44	3.5E−04	1312	—
		9	−RTS	L	Thalamus—(VL)	−14	−18	2	3.66	1.0E−03	200	—
	Left	1	ET > HV	R	Middle occipital gyrus	32	−83	26	4.49	7.5E−05	712	—
		2	ET > HV	L	Cuneus	−5	−93	36	4.03	2.9E−04	440	—
		3	ET < HV	R	Thalamus—(VL)	16	−9	4	4.45	8.4E−05	520	—
		4	ET < HV	R	Supramarginal gyrus	48	−31	25	4.25	1.4E−04	832	0.0121
		5	ET < HV	R	Precentral gyrus (M1)	20	−27	46	4.03	2.8E−04	1512	0.0067
		5b	ET < HV	R	Postcentral gyrus (SMA)	26	−33	54	3.91	4.0E−04		0.0068
		6	ET < HV	L	Cerebellum IX	−9	−49	−33	3.71	7.1E−04	48	—
		7	+LTS	R	Anterior insula	39	21	7	4.03	8.7E−04	1512	—
		8	−LTS	L	Precentral gyrus	−50	−5	50	3.64	1.0E−03	264	—
MVPA (L9)	Right	1	ET < HV	R	Supramarginal gyrus	41	−30	25	4.84	2.6E−05	864	—
		2	ET < HV	L	pSTG	−62	−32	13	4.07	2.5E−04	312	—
		3	ET < HV	L	Cuneus	−14	−78	39	3.73	6.6E−04	20	—
		4	−RTS	R	Postcentral gyrus (SMA)	6	−5	56	4.26	1.5E−04	1000	—
		5	−RTS	R	Cerebellum crus I	46	−65	−25	3.48	9.9E−04	120	—
	Left	1	ET > HV	R	Posterior cingulate gyrus	5	−46	9	4.63	4.0E−05	4000	—
		2	ET > HV	R	Angular gyrus	59	−50	21	4.26	1.4E−04	552	—
		3	ET > HV	R	Inferior occipital gyrus	55	−58	1	3.87	4.4E−04	192	—
		4	ET < HV	R	Supramarginal gyrus	43	−32	23	5.06	1.4E−05	2880	0.0043
		5	ET < HV	R	Thalamus—(VL)	15	−16	0	3.68	7.7E−04	976	—
		6	ET < HV	R	Putamen	27	5	3	3.66	8.1E−04	352	—
		7	+LTS	R	Supramarginal gyrus	52	−27	31	4.09	2.0E−04	1040	—
		8	+LTS	R	Insula	40	15	7	5.3	6.0E−06	2040	0.0223
		9	+LTS	L	Insula	−42	4	6	4.34	1.0E−04	680	0.0203
		10	+LTS	L	Cerebellum VIII–IX	−17	−51	−47	3.94	4.0E−04	96	—

Abbreviations: ET, Essential tremor; HV, Healthy volunteers; L, Left; L9, lag 9; LTS, left tremor severity; M1, primary motor cortex; *N*, Cluster number; pSTG, Posterior superior temporal gyrus; R, Right; RTS, right tremor severity; SMA, supplementary motor area; VL, ventral lateral.

For the right finger‐tapping task, ET patients showed decreased BOLD responses in both thalami (*t* = 5.72, *p*_*TFCE*_ = 0.003) and the right supramarginal gyrus (*t = 4.49*, *p*_*TFCE*_ = 0.017) compared to healthy volunteers. Additionally, in ET patients, higher RTS was associated with increased BOLD responses in the left cerebellum IX (*t* = 5.01, *p*_*unc*_ = 1.0E−04) and right postcentral gyrus (*t* = 4.45, *p*_*unc*_ = 3.6E−04), whereas higher RTS was associated with decreased BOLD responses in the left precentral gyrus (*t* = 4.44, *p*_*unc*_ = 3.5E−04) and the left ventral lateral thalamus (*t* = 3.66, *p*_*unc*_ = 0.001).

During the left finger‐tapping task, ET patients exhibited decreased BOLD responses in the right supramarginal gyrus (*t* = 4.25, *p*_*TFCE*_ = 0.012), the right precentral gyrus (*t* = 4.45, *p*_*TFCE*_ = 8.4E−05), and the right thalamus at a more liberal, uncorrected threshold (*t* = 4.25, *p*_*unc*_ = 0.012) compared to healthy volunteers. In ET patients, higher LTS was associated with increased BOLD responses in the right anterior insula (*t* = 4.03, *p*_*unc*_ = 8.7E−04), whereas higher LTS was linked to decreased BOLD responses in the left precentral gyrus (*t* = 3.64, *p*_*unc*_ = 1.0E−03). An exploratory whole‐brain Group × Hand interaction analysis (ET vs. HV × Right vs. Left hand) did not reveal any statistically significant effects after TFCE correction.

### 3.3 MVPA Results

MVPA results revealed decoding performance differences within and between groups. Decoding performance was evaluated across all predefined poststimulus lags (3, 5, 7, and 9 s). Robust and consistent effects were observed at lag 9, whereas effects at earlier lags were weaker. Accordingly, the main results are reported for lag 9, with results for all other lags provided in Table S1.

**FIGURE 4:**
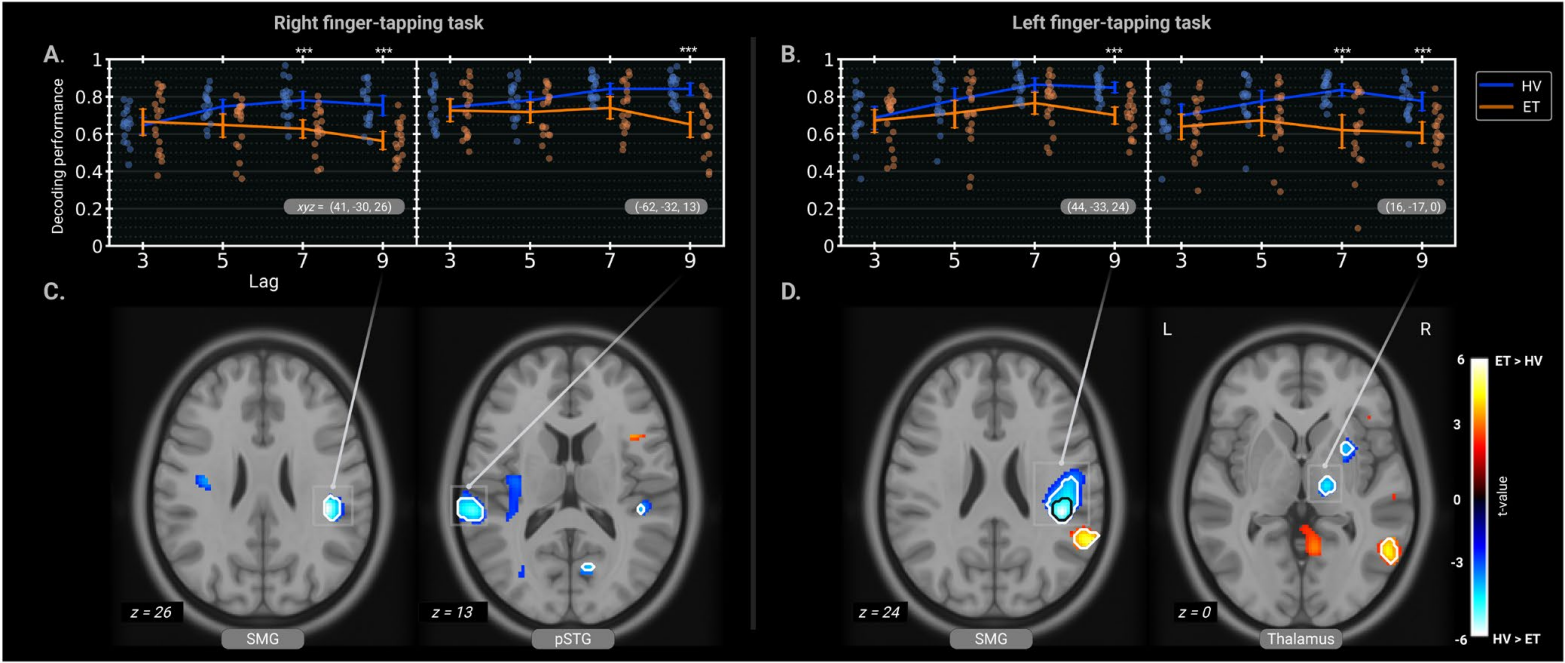
(A) Right‐ and (B) Left‐finger‐tapping task distribution of local maxima in decoding performance; blue for healthy volunteers and red for ET across time‐lags, error bars represent confidence interval of 95% (***p_*unc*_ < 0.001). (C) Right‐ and (D) Left‐finger‐tapping task local maxima identified by MVPA analysis at lag 9, thresholded at *p*_*unc*_ < 0.001 (white contour) and TFCE corrected *p*_*TFCE*_ < 0.05 (black contour); blue colors represent areas where ET patients exhibited decreased decoding performance compared to healthy volunteers (HV), while red colors indicate areas where ET demonstrated greater relevance than healthy volunteers.

Within‐group findings are shown in Figure S4. Between‐group results for both finger‐tapping tasks appear in Table 3 and Figure 4, with tremor severity associations in Figure 3C,D.

For the right finger‐tapping task, ET patients showed decreased decoding performance in the right supramarginal gyrus (*t* = 4.84, *p*_*unc*_ = 2.6E−05), left posterior superior temporal gyrus (*t* = 4.07, *p*_*unc*_ = 2.5E−04), and left cuneus (*t* = 3.73, *p*_*unc*_ = 6.6E−04) compared to healthy volunteers. In ET patients, higher RTS was associated with decreased decoding performance in the right postcentral gyrus (*t* = 4.26, *p*_*unc*_ = 1.0E−04) and right cerebellum Crus I (*t* = 3.48, *p*_*unc*_ = 9.9E−04).

During the left finger‐tapping task, ET patients showed increased decoding performance in the right posterior cingulate gyrus (*t* = 4.63, *p*_*unc*_ = 4.9E−05), right angular gyrus (*t* = 4.26, *p*_*unc*_ = 1.4E−04), and right inferior occipital gyrus (*t* = 3.87, *p*_*unc*_ = 4.4E−04) compared to healthy volunteers. Additionally, ET patients exhibited lower decoding performance in the right supramarginal gyrus (*t* = 5.06, *p*_*TFCE*_ = 0.004), right thalamus (*t* = 3.68, *p*_*unc*_ = 7.7E−04), and right putamen (*t* = 3.66, *p*_*unc*_ = 8.1E−04) compared to healthy volunteers. In ET patients, higher LTS was associated with increased decoding performance in the right supramarginal gyrus (*t* = 4.09, *p*_*unc*_ = 2.0E−04), right insula (*t* = 5.3, *p*_*TFCE*_ = 0.022), left insula (*t* = 4.34, *p*_*TFCE*_ = 0.020), and left cerebellum VIII–IX (*t* = 3.94, *p*_*unc*_ = 4.0E−04).

## 4 Discussion

Using task‐based fMRI with GLM and MVPA, we examined brain network differences between right‐ and left‐hand motor movements in ET patients versus healthy volunteers. Our findings support the involvement of the cerebello‐thalamo‐cortical network, with lateralization emerging as an important feature of task‐related motor network engagement in ET. Below, we describe these patterns across the cerebellum, thalamus, and cortical areas.

As the Group × Hand interaction did not reach statistical significance, the lateralization patterns described below should be interpreted as task‐related differences associated with Hand and overall Group effects, rather than evidence for altered hand‐specific lateralization in ET.

In the cerebellum, during the right finger‐tapping task, ET patients exhibited increased BOLD responses in the left cerebellum (Crus I) at an uncorrected threshold, suggesting altered task‐related recruitment. This finding aligns with a previous study that reported elevated cerebellar activity in ET patients during a postural task when provoking tremor, compared to healthy volunteers mimicking such tremor [8]. However, greater RTS was linked to reduced decoding in the right Crus I, suggesting lateralized processing inefficiency. While both hemispheres were active, right Crus I impairment appeared proportional to tremor severity, possibly reflecting compensatory yet inefficient motor network engagement rather than effective adaptation [32].

For the left finger‐tapping task, ET patients showed reduced BOLD responses in the left cerebellum (lobule IX), consistent with ipsilateral motor activation. Higher LTS was linked to increased decoding in lobules VIII–IX, suggesting tremor severity is associated with increased recruitment of these regions. These lobules are key for coordinating smooth movement [33]. The increased decoding performance in left lobules VIII–IX may indicate greater task‐related recruitment of cerebellar regions supporting motor coordination in individuals with higher tremor severity.

The cerebellum in ET has been linked to disruptions in the cerebello‐thalamo‐cortical connectivity [34], and microstructural changes [16]. In our study, cerebellar activity varied by task, showing lateralized activation patterns influenced by tremor severity. Crus I was bilaterally engaged during right‐hand tapping, while reduced lobule IX activation during left‐hand tasks was accompanied by compensatory recruitment of lobules VIII–IX. These findings suggest task‐specific cerebellar specialization and potential adaptive engagement in ET.

In the thalamus during the right finger‐tapping task, a bilateral decrease in thalamic BOLD responses in ET patients was found compared to healthy volunteers, including involvement of the pulvinar and right mediodorsal nucleus, which has not been previously reported in motor‐task fMRI studies. However, previous research has shown significant biochemical and metabolic alterations bilaterally in the thalamic volumes of ET patients, particularly concerning increased levels of glutamate and glutamine [35]. While both thalami showed decreased BOLD responses in ET, only the VL of the left thalamus, which is classically implicated in motor circuitry [36], demonstrated a significant negative correlation with the tremor severity at an uncorrected threshold, reflecting that the more severe a patient's tremor, the lower the activity in the VL of the left thalamus. These findings support a more prominent contralateral contribution of the thalamus to task‐related motor network alterations associated with tremor severity [37].

For the left finger‐tapping task, the VL of the right thalamus was found to have decreased BOLD responses and showed lower decoding performance at an uncorrected threshold in ET patients compared to healthy volunteers. Tremor‐related activity in the VL has previously been reported during voluntary movements and shows higher firing rates in ET than in Parkinson's disease [38]. Moreover, VIM deep brain stimulation (DBS), targeting this region, is highly effective for tremor reduction [39], with stronger contralateral connectivity associated with better outcomes [37]. While VL hyperactivity is reported at rest, our findings suggest reduced activation during movement, likely due to task interference. A similar reduction has been observed during writing tasks [12].

These findings further support the thalamus as a central relay within the cerebello‐thalamo‐cortical network. Nicoletti et al. reported that in ET the thalamus presented disrupted connectivity within motor pathways [17]. Our findings reveal a disrupted, lateralized thalamic pattern in ET. During right‐hand tasks, both thalami showed reduced activity, but only the left VL correlated with tremor severity, supporting a contralateral role. In left‐hand tasks, the right VL showed decreased activation and decoding performance, further implicating contralateral VL dysfunction in tremor.

In the cortical regions during the right finger‐tapping task, ET patients exhibited decreased BOLD responses and lower decoding performance in the right supramarginal gyrus compared to healthy volunteers. The right supramarginal, particularly its anterior segment, is implicated in proprioception and higher‐order somatosensory processing contributing to motor control, with dominance of the right hemisphere [40, 41]. Given its key role in cerebellar‐parietal interactions, which integrate sensory feedback and motor predictions [42], its disruption suggests a link between ET‐related motor dysfunction and proprioceptive deficits, particularly in the right hemisphere.

For the left finger‐tapping task, ET patients showed reduced BOLD responses and decoding performance in the right supramarginal gyrus—mirroring its involvement in the right‐hand task. A positive correlation with tremor severity at an uncorrected threshold suggests more pronounced dysfunction with increasing tremor burden. The consistent involvement of the right supramarginal gyrus across both hand conditions suggests a hand‐independent role in ET‐related motor network alterations. Resting‐state fMRI studies have linked this region, along with the right postcentral gyrus, to disrupted motor network dynamics and tremor relief following focused ultrasound thalamotomy [43, 44]. Importantly, convergent evidence from our NEMO FDG‐PET study in largely overlapping patients demonstrated decreased glucose metabolism in the right inferior parietal lobule (peak MNI: 52, −44, 54), corresponding anatomically to the right supramarginal gyrus, further supporting involvement of this region in ET pathophysiology [45]. The convergence of task‐based fMRI, resting‐state connectivity, and metabolic PET findings across modalities strengthens the evidence that the inferior parietal lobule is consistently involved in essential tremor. The supramarginal gyrus has been implicated in monitoring mismatches between intended and executed movements and shows abnormal engagement in functional (conversion) tremor [46]. Although ET and functional tremor differ fundamentally in etiology, altered supramarginal engagement in the present study may reflect disrupted sensorimotor integration and impaired monitoring of voluntary motor commands in the presence of involuntary tremor. Together, these multimodal findings suggest that altered sensorimotor integration within the inferior parietal lobule constitutes an important component of essential tremor pathophysiology, extending beyond the classical cerebello‐thalamo‐cortical circuit.

Higher tremor severity also correlated with greater BOLD responses in the right insula and higher decoding performance in both insulae. Reduced regional homogeneity in the insula has been associated with increased tremor severity [47]. Its function in integrating sensory, motor, and autonomic information [48] suggests that insula engagement during motor execution scales with tremor severity, particularly during left‐hand motor tasks.

Together, these findings indicate a disrupted and lateralized pattern of cortical involvement in ET, extending beyond primary motor regions to higher‐order sensorimotor integration areas. Task‐specific alterations in the supramarginal gyrus and insula highlight the contribution of distributed cortical networks to motor network alterations associated with tremor severity, rather than isolated tremor physiology.

### 4.1 Strengths and Limitations

Including both hands in our task design allowed a detailed assessment of brain lateralization in ET. A key strength was the use of strict inclusion criteria, ensuring diagnostic accuracy. While the sample size limited statistical power, a more liberal threshold enabled broader comparisons, albeit with reduced spatial specificity. However, the modest sample size limited statistical power, particularly for detecting subtle interaction effects and small effect sizes. As a result, some findings, especially those observed at uncorrected thresholds, should be interpreted cautiously and considered exploratory.

Importantly, no objective behavioral performance measures (e.g., tapping frequency, error rates) or physiological recordings (e.g., accelerometry or EMG) were acquired during scanning. Consequently, voluntary motor execution and superimposed tremor‐related activity cannot be disentangled, and the reported neural patterns should be interpreted as reflecting motor execution in the presence of tremor. Moreover, tremor asymmetry was assessed using hand‐specific Fahn–Tolosa–Marin scores, which, while clinically validated, lack the temporal precision of accelerometry or EMG for quantifying subtle inter‐hand differences during task performance.

Combining GLM and MVPA strengthened our results. While GLM detects overall BOLD changes [49], MVPA captured subtle, distributed patterns—particularly in the insula—often missed by GLM [20]. It also enabled time‐lag analysis, where reduced decoding at longer lags in ET patients suggested difficulties sustaining motor patterns, consistent with known visuomotor adaptation deficits and disrupted motor networks, especially in the nondominant hand [34, 50].

Our study also addressed individual variability by linking tremor severity to neural activity per hand. Stronger correlations during left‐hand tasks likely reflect the higher proportion of patients with left‐sided tremor. These findings underscore the need to further explore ET heterogeneity and the role of tremor asymmetry in brain function.

## 5 Conclusions

This study reveals that the thalamus, cerebellum, supramarginal gyrus, and insula are key contributors to motor network alterations associated with tremor severity in ET, with lateralization emerging as an important organizing principle. Task‐related engagement of cerebellar regions scaled with tremor severity, consistent with possible compensatory recruitment at the neural level, while insular involvement reflected tremor‐severity–related differences in task‐related network organization.

Distinct patterns of lateralization were observed: right‐hand tapping engaged bilateral motor circuits, whereas left‐hand tapping showed stronger right‐lateralized involvement, particularly within the thalamus, supramarginal gyrus, insula, and cerebellum.

These findings underscore the relevance of lateralized brain dynamics in ET and support further investigation of hand‐specific, network‐level mechanisms, ideally incorporating concurrent behavioral and physiological measures to inform future therapeutic strategies.

## Author Contributions

**Jelle R. Dalenberg:** methodology, writing – review and editing, conceptualization, investigation, supervision, software, data curation, validation, resources. **Remco J. Renken:** writing – review and editing, methodology, supervision, validation. **A. M. Madelein van der Stouwe:** data curation, writing – review and editing, methodology, validation, investigation, supervision, conceptualization, resources. **Alma S. Torres‐Torres:** conceptualization, methodology, visualization, writing – original draft, formal analysis, software. **Bauke M. de Jong:** conceptualization, writing – review and editing, supervision. **Marina A. J. Tijssen:** writing – review and editing, funding acquisition, project administration, supervision, conceptualization, resources. **Giorgia Sciacca:** validation, data curation.

## Funding

This work was supported by ZonMw‐TOP 2018/91218013.

## Conflicts of Interest

The authors declare no conflicts of interest.

## Supporting information


**Figure S1:** Experimental paradigm of one block that includes right and left finger‐tapping task (FTT) and rest, this block was repeated five times.
**Figure S2:** Asymmetry between left tremor severity (LTS) and right tremor severity (RTS) in ET patients, as measured by the Fahn‐Tolosa‐Marin Tremor Rating Scale A (FTM‐TRS‐A). Each point, connected by lines, represents an individual patient's scores. Green lines indicate patients with greater LTS than RTS (50%), orange lines represent patients with equal tremor severity in both hands (22%), and blue lines show patients with greater RTS than LTS (28%). The red line represents the mean difference between LTS and RTS (*p* = 0.23).
**Figure S3:** (A) Right‐ and (B) Left‐finger‐tapping task the within‐group GLM contrast for healthy volunteers (healthy volunteers) and essential tremor (ET) were both thresholded at a *p*_*unc*_ < 0.001, corrected with *p*_*TFCE*_ < 0.05.
**Figure S4:** (A) Right‐ and (B) Left‐finger‐tapping task the within‐group MVPA contrast for healthy volunteers (healthy volunteers) and essential tremor (ET) were both thresholded at a *p*_*unc*_ < 0.001, corrected with *p*_*TFCE*_ < 0.05.
**Table S1:** MVPA between‐group comparison of brain regions in right‐ and left‐finger‐tapping task per lag.

## Data Availability

The data that support the findings of this study are available from the corresponding author upon reasonable request.
