# Supplementary material for: Hand‐Specific Engagement of Cerebello–Thalamo–Cortical and Higher‐Order Sensorimotor Networks in Essential Tremor: Converging Evidence From GLM and MVPA‐Based fMRI Analysis
Source: Eur J Neurol. 2026 Jun 14;33(6):e70662. doi: 10.1111/ene.70662 (PMC13266084; doi:10.1111/ene.70662)
Supplement: Supplementary file 1 — Figure S1: Experimental paradigm of one block that includes right and left finger‐tapping task (FTT) and rest, this block was repeated five times. Figure S2: Asymmetry between left tremor severity (LTS) and right tremor severity (RTS) in ET patients, as measured by the Fahn‐Tolosa‐Marin Tremor Rating Scale A (FTM‐TRS‐A). Each point, connected by lines, represents an individual patient's scores. Green lines indicate patients with greater LTS than RTS (50%), orange lines represent patients with equal tremor severity in both hands (22%), and blue lines show patients with greater RTS than LTS (28%). The red line represents the mean difference between LTS and RTS (p = 0.23). Figure S3: (A) Right‐ and (B) Left‐finger‐tapping task the within‐group GLM contrast for healthy volunteers (healthy volunteers) and essential tremor (ET) were both thresholded at a p unc < 0.001, corrected with p TFCE < 0.05. Figure S4: (A) Right‐ and (B) Left‐finger‐tapping task the within‐group MVPA contrast for healthy volunteers (healthy volunteers) and essential tremor (ET) were both thresholded at a p unc < 0.001, corrected with p TFCE < 0.05. Table S1: MVPA between‐group comparison of brain regions in right‐ and left‐finger‐tapping task per lag. [file ENE-33-e70662-s001.docx]

# **Supplementary material**


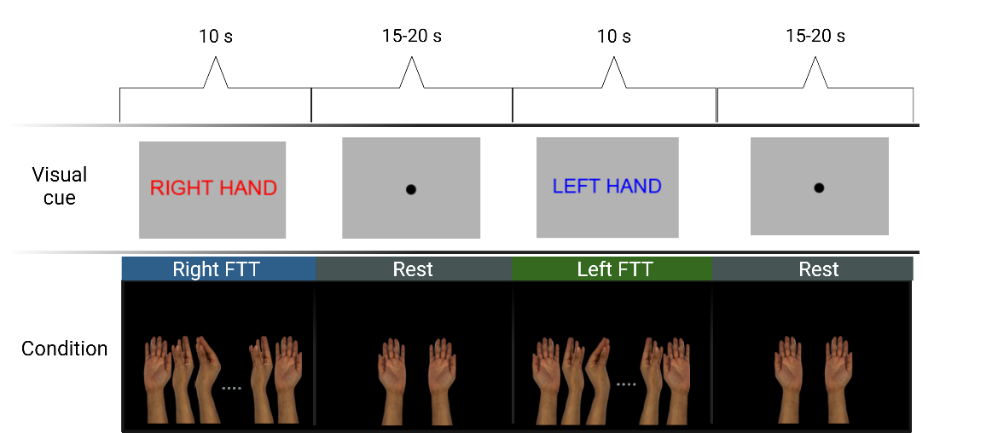


**Supplementary Figure 1**. Experimental paradigm of one block that includes right and left finger-tapping task (FTT) and rest, this block was repeated five times.


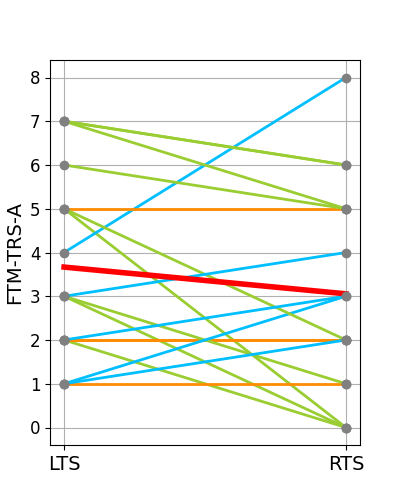


**Supplementary Figure 2**. Asymmetry between left tremor severity (LTS) and right tremor severity (RTS) in ET patients, as measured by the Fahn-Tolosa-Marin Tremor Rating Scale A (FTM-TRS-A). Each point, connected by lines, represents an individual patient's scores. Green lines indicate patients with greater LTS than RTS (50%), orange lines represent patients with equal tremor severity in both hands (22%), and blue lines show patients with greater RTS than LTS (28%). The red line represents the mean difference between LTS and RTS (p = 0.23).


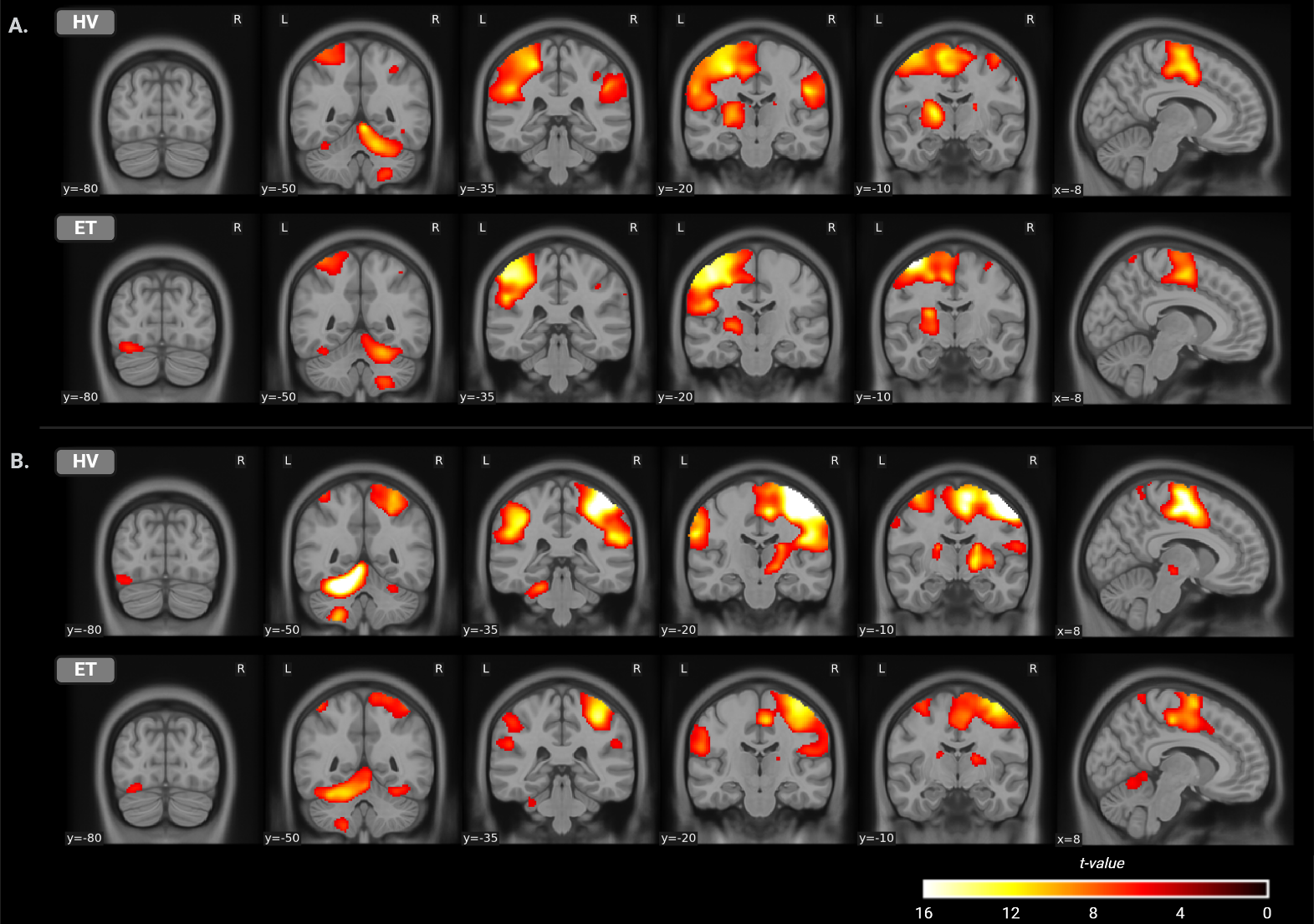


**Supplementary Figure 3**. A) Right- and B) Left- finger-tapping task the within-group GLM contrast for healthy volunteers (healthy volunteers) and essential tremor (ET) were both thresholded at a p_unc_ < 0.001, corrected with p_TFCE_ < 0.05.

*
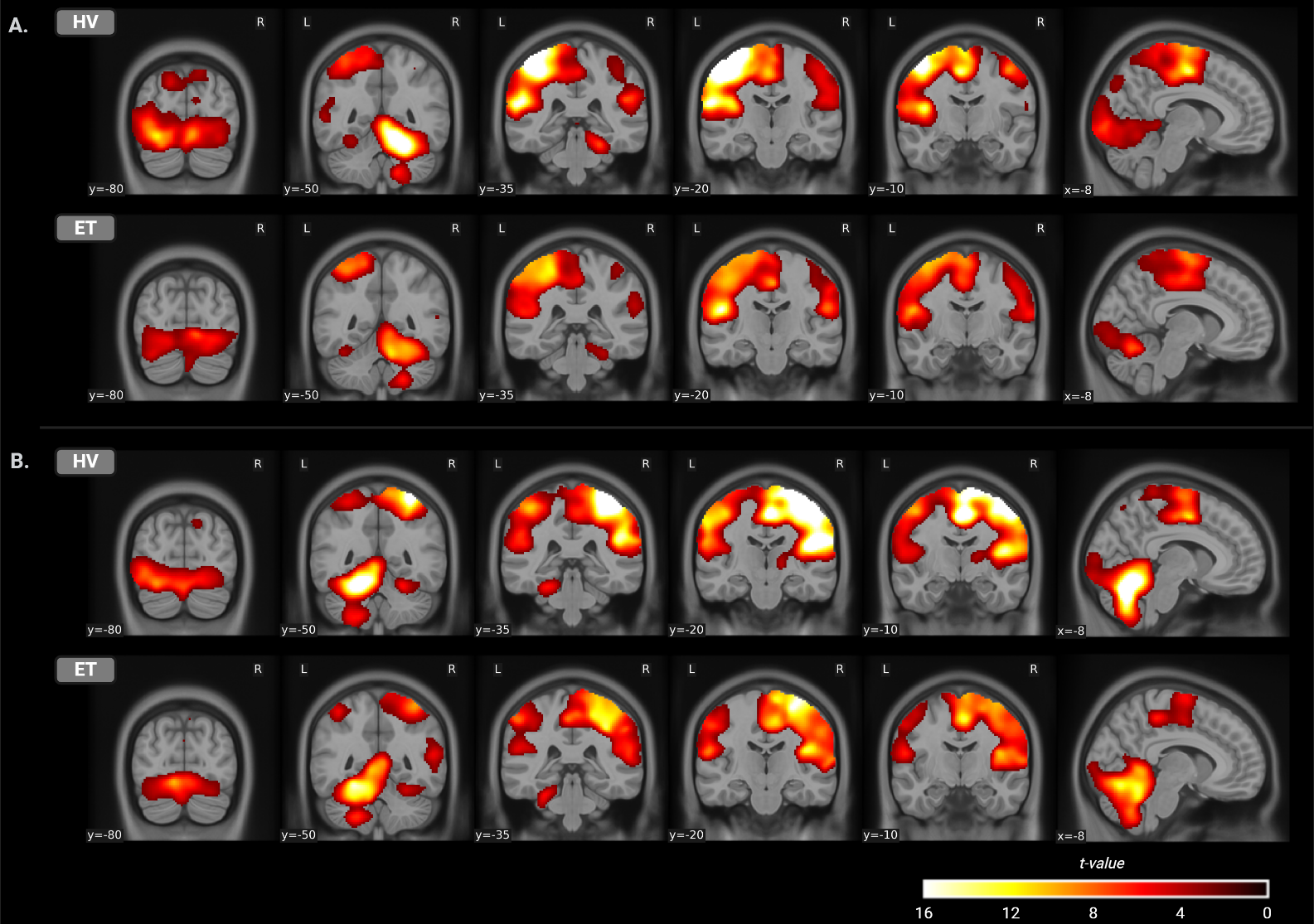
*

**Supplementary Figure 4.** A) Right- and B) Left- finger-tapping task the within-group MVPA contrast for healthy volunteers (healthy volunteers) and essential tremor (ET) were both thresholded at a punc < 0.001, corrected with pTFCE < 0.05.

**Supplementary Table 1**. MVPA between-group comparison of brain regions in right- and left- finger-tapping task per lag.

|  | **Finger**  **tapping task** | **N** | **Contrasts** |  | **Brain region** | **X** | **Y** | **Z** | **t-value** | **p_unc_** | **Cluster Size** [mm^3^] | **p_TFCE_** |
| --- | --- | --- | --- | --- | --- | --- | --- | --- | --- | --- | --- | --- |
| L3 |  |  |  |  |  |  |  |  |  |  |  |  |
|  | Right |  |  |  |  |  |  |  |  |  |  |  |
|  |  | 1 | ET < HV | L | Inferior temporal gyrus | -36 | -22 | -20 | 4.28 | 1.3E-04 | 368 | _ |
|  | Left |  |  |  |  |  |  |  |  |  |  |  |
|  |  | 1 | ET > HV | R | Parahippocampal gyrus | 7 | -10 | -22 | 4.17 | 7.5E-05 | 160 | __ |
| L5 |  |  |  |  |  |  |  |  |  |  |  |  |
|  | Right |  |  |  |  |  |  |  |  |  |  |  |
|  |  | 1 | ET > HV | R | Middle frontal gyrus | 43 | 47 | 19 | 4.52 | 6.7E-05 | 656 | _ |
|  |  | 2 | ET > HV | R | Superior frontal gyrus | 43 | 23 | 35 | 4.42 | 9.0E-05 | 160 | _ |
|  | Left |  |  |  |  |  |  |  |  |  |  |  |
|  |  | 1 | ET > HV | R | Inferior frontal gyrus | 29 | 35 | -6 | 4.31 | 1.2E-04 | 328 | _ |
|  |  | 2 | ET < HV | L | Superior frontal gyrus | 0 | 49 | 41 | 3.89 | 4.2E-04 | 104 | _ |
| L7 |  |  |  |  |  |  |  |  |  |  |  |  |
|  | Right |  |  |  |  |  |  |  |  |  |  |  |
|  |  | 1 | ET > HV | R | Middle temporal gyrus | 40 | 30 | -25 | 4.95 | 1.8E-05 | 704 | _ |
|  |  | 2 | ET < HV | L | Superior temporal gyrus | -39 | -27 | 10 | 4.03 | 2.8E-04 | 240 | _ |
|  | Left |  |  |  |  |  |  |  |  |  |  |  |
|  |  | 1 | ET > HV | R | Inferior frontal gyrus | 28 | 37 | -9 | 4.37 | 1.0E-04 | 408 | _ |
|  |  | 2 | ET > HV | L | Inferior frontal gyrus | -34 | 27 | -15 | 3.95 | 3.5E-04 | 128 | _ |
|  |  | 3 | ET < HV | L | Inferior temporal gyrus | -52 | -30 | -16 | 4.38 | 1.0E-04 | 968 | _ |
| L9 |  |  |  |  |  |  |  |  |  |  |  |  |
|  | Right |  |  |  |  |  |  |  |  |  |  |  |
|  |  | 1 | ET < HV | R | Supramarginal gyrus | 41 | -30 | 25 | 4.84 | 2.6E-05 | 864 | _ |
|  |  | 2 | ET < HV | L | pSTG | -62 | -32 | 13 | 4.07 | 2.5E-04 | 312 | _ |
|  |  | 3 | ET < HV | L | Cuneus | -14 | -78 | 39 | 3.73 | 6.6E-04 | 20 | _ |
|  | Left |  |  |  |  |  |  |  |  |  |  |  |
|  |  | 1 | ET > HV | R | Posterior cingulate gyrus | 5 | -46 | 9 | 4.63 | 4.0E-05 | 4000 | _ |
|  |  | 2 | ET > HV | R | Angular gyrus | 59 | -50 | 21 | 4.26 | 1.4E-04 | 552 | _ |
|  |  | 3 | ET > HV | R | Inferior occipital gyrus | 55 | -58 | 1 | 3.87 | 4.4E-04 | 192 | _ |
|  |  | 4 | ET < HV | R | Supramarginal gyrus | 43 | -32 | 23 | 5.06 | 1.4E-05 | 2880 | 0.0043 |
|  |  | 5 | ET < HV | R | Thalamus – (VL) | 15 | -16 | 0 | 3.68 | 7.7E-04 | 976 | _ |
|  |  | 6 | ET < HV | R | Putamen | 27 | 5 | 3 | 3.66 | 8.1E-04 | 352 | _ |
| Cluster number (N), Essential tremor (ET), Healthy volunteers (HV), Right (R), Left (L), right tremor severity (RTS), left tremor severity (LTS), Posterior superior temporal gyrus (pSTG) supplementary motor area (SMA) and primary motor cortex (M1), ventral lateral (VL), lag 3 (L3), lag 5 (L5), lag 7 (L7) and lag 9 (L9). | | | | | | | | | | | | |
